# AI-based diabetes care: risk prediction models and implementation concerns

**DOI:** 10.1038/s41746-024-01034-7

**Published:** 2024-02-15

**Authors:** Serena C. Y. Wang, Grace Nickel, Kaushik P. Venkatesh, Marium M. Raza, Joseph C. Kvedar

**Affiliations:** Harvard Medical School, Boston, MA USA

**Keywords:** Preventive medicine, Technology

## Abstract

The utilization of artificial intelligence (AI) in diabetes care has focused on early intervention and treatment management. Notably, usage has expanded to predict an individual’s risk for developing type 2 diabetes. A scoping review of 40 studies by Mohsen et al. shows that while most studies used unimodal AI models, multimodal approaches were superior because they integrate multiple types of data. However, creating multimodal models and determining model performance are challenging tasks given the multi-factored nature of diabetes. For both unimodal and multimodal models, there are also concerns of bias with the lack of external validations and representation of race, age, and gender in training data. The barriers in data quality and evaluation standardization are ripe areas for developing new technologies, especially for entrepreneurs and innovators. Collaboration amongst providers, entrepreneurs, and researchers must be prioritized to ensure that AI in diabetes care is providing quality and equitable patient care.

## Introduction

With the urgent need to address the increasing incidence and prevalence of diabetes globally, promising new applications of artificial intelligence (AI) for this chronic disease have arisen—development of predictive models, risk stratification, evaluation of novel risk predictors, and therapeutic management.

Thus far, most FDA-approved AI tools have been designed for early intervention and treatment management. Several of these tools are currently used in clinical diabetes care. For early intervention, in 2018 the FDA approved the autonomous AI system Digital Diagnostics, which was found to have high diagnostic accuracy in recognizing diabetes retinopathy in retinal screening images^1^. In the same year, the FDA approved the Guardian Connect System, which uses AI technology to interpret biomedical data and predict a hypoglycemic attack an hour in advance^2^. Since then, the FDA has also approved AI technologies that assist with optimizing insulin dosing and therapy for patients^3,4^.

## Risk prediction

Beyond intervention and treatment, AI is now being utilized to predict an individual’s risk for developing type 2 diabetes (T2DM) and potential complications. Identifying high-risk individuals and personalizing prevention strategies and targeted treatments could delay or prevent the onset of diabetes and future health complications.

Mohsen et al. conducted a scoping review of 40 studies that investigated AI-based models for diabetes risk prediction^5^. The performance of the algorithms was measured using the area under the curve (AUC) metric in most studies. AUC is a commonly used metric in machine learning to determine the performance of AI models; an AUC value of 1 indicates a perfect model. Of these models, most were classical machine learning models with electronic health records as the predominant data source. Although a modest number (*n* = 10) studies adopted multimodal approaches (combining EHR with multi-omics or medical imaging data), these were shown to be superior to unimodal models (*n* = 30). For example, one multimodal approach revealed that a model combining genomic, metabolomic, and clinical risk factors was superior in predicting T2DM (AUC of 0.96) compared to a genomics-only model (AUC of 0.586) and clinical-only model (AUC of 0.798)^6^. Improved performance of multimodal models can be attributed to integrating multiple types of data such as clinical, genetic, and biomarker data, providing a comprehensive view of an individual’s health status.

However, developing multimodal models is extremely time-consuming and as a result, it is challenging to quickly and easily scale such models. Merging data sources can complicate the understanding of interactions among modalities and the rationale behind predictions, resulting in a scarcity of multimodal AI models for T2DM. Still, in light of promising outcomes from multimodal models for chronic conditions such as T2DM, there is a growing effort to create more individualized multimodal virtual representations of patients, also known as digital twins. A digital twin is created using multimodal individual patient data, population data, and real-time input of patient and environmental variables^7^.

## Implementation concerns

While the review by Mohsen et al. suggests promising AI technologies for T2DM risk prediction, the results should be approached with caution. Determining the best-performing model is challenging, given that the type and combination of input risk predictors for diabetes (e.g., BMI, waist circumference, fasting plasma glucose, age, alcohol intake, blood pressure, etc.) can influence the model’s performance. For example, the XGBoost algorithm—a machine learning algorithm that uses a sequentially iterative process to learn and correct previous errors—was used in three unimodal studies^8^. Although the same algorithm was implemented, each study used different risk predictors and datasets, which resulted in widely disparate AUC values of 0.91, 0.83, and 0.67^9–11^. Even with multimodal models, direct comparisons across studies must be considered carefully due to biases such as variations in datasets, evaluation metrics, and prediction horizons.

There are further concerns with bias due to the variability of demographic representation across models, with many in this study showing a pronounced imbalance of gender, ethnicity, and age. Only five studies provided insights into calibration of models and only five conducted external validations. Most studies did not evaluate the algorithm’s performance across different demographic groups or use calibration and fairness (e.g., demographic parity, equal opportunity) metrics to assess disparities in predictions across these groups^12,13^. T2DM is a multifactorial disease impacted by biological, clinical, and socioeconomic factors. As Black and Mexican Americans have an increased prevalence of diabetes compared to their white counterparts, the omission of certain demographics in training models could perpetuate existing health inequities for already at-risk populations and introduce a high probability of bias^14^.

To ensure demographic representation in datasets, it is necessary to implement policies that require mandatory representation criteria for approval and adoption. It is important to integrate appropriate evaluation metrics, such as using Quality Assessment of Diagnostic Accuracy Studies (QUADAS) AI frameworks to evaluate a model’s risk of bias^15^. External validation is also crucial to ensure models can generalize beyond specific datasets used for training. The QUADAS AI tool is an evidence-based tool designed to assess the risk of bias—related to patient selection, diagnostic test interpretation, and choice of reference standard—and applicability—generalizability of a study’s findings to the intended population—of diagnostic accuracy studies that involve AI. A comprehensive approach will ensure that equitable and unbiased AI models are used to prevent exacerbating existing health disparities.

## On the horizon

AI tools in diabetes care, specifically those trained with a multimodal approach, have promising applications in risk prediction. However, since unimodal approaches are still more commonplace, there remains untapped potential in this field to use more accurate tools that meet the caliber of clinical care patients deserve. Novel solutions are necessary on two fronts—data quality and standardized evaluation metrics. Comprehensive and diverse data sets to train models are necessary to create accurate tools. Especially as health data is continuously collected to create robust datasets, it is important to organize and structure the data for potential compatibility and interoperability for developing multimodal algorithms^16^. Universal evaluation protocols are also necessary to mitigate the propagation of health inequities. The rapid and largescale adoption of AI in healthcare cannot occur before the problems in data quality and bias are addressed—making these two fronts ripe areas of development for innovations and new technologies from the private sector. Solutions to encourage collaboration and transparency on these two fronts could be inspired by structures in other AI fields, such as open-source platforms, ethical review processes, and enforcement of bias testing to uphold a higher standard of practice. To ensure patient care lies at the center of novel AI tools in diabetes care, solutions must be rooted in collaborative efforts with all stakeholders—clinicians, researchers, policymakers, and entrepreneurs—as we continue to innovate in the field of AI and diabetes.
